# Extracellular Vesicles for the Treatment of Radiation Injuries

**DOI:** 10.3389/fphar.2021.662437

**Published:** 2021-05-18

**Authors:** Lalitha Sarad Yamini Nanduri, Phaneendra K. Duddempudi, Weng-Lang Yang, Radia Tamarat, Chandan Guha

**Affiliations:** ^1^ Department of Radiation Oncology, Albert Einstein College of Medicine, Montefiore Medical Center, New York, NY, United States; ^2^ Department of Biochemistry, Albert Einstein College of Medicine, Montefiore Medical Center, New York, NY, United States; ^3^ Institut de Radioprotection et de Sûreté Nucléaire (IRSN), Fontenay-aux-Roses, France; ^4^ Department of Pathology, Albert Einstein College of Medicine, Montefiore Medical Center, New York, NY, United States; ^5^ Department of Urology, Albert Einstein College of Medicine, Montefiore Medical Center, New York, NY, United States; ^6^ Institute for Onco-Physics, Albert Einstein College of Medicine, Montefiore Medical Center, New York, NY, United States

**Keywords:** acute radiation syndrome, radio mitigation, medical countermeasures against radiation, mesenchymal stromal/stem cells, endothelial cells, macrophages, extracellular vesicles, radiation injuries

## Abstract

Normal tissue injury from accidental or therapeutic exposure to high-dose radiation can cause severe acute and delayed toxicities, which result in mortality and chronic morbidity. Exposure to single high-dose radiation leads to a multi-organ failure, known as acute radiation syndrome, which is caused by radiation-induced oxidative stress and DNA damage to tissue stem cells. The radiation exposure results in acute cell loss, cell cycle arrest, senescence, and early damage to bone marrow and intestine with high mortality from sepsis. There is an urgent need for developing medical countermeasures against radiation injury for normal tissue toxicity. In this review, we discuss the potential of applying secretory extracellular vesicles derived from mesenchymal stromal/stem cells, endothelial cells, and macrophages for promoting repair and regeneration of organs after radiation injury.

## Introduction

The first report of detrimental effects of ionizing radiation on healthy normal tissues came to light after the atomic bomb explosions in 1945. This unprecedented event introduced to the world the lethal radiation poisoning or sickness, also known as acute radiation syndrome (ARS), where a relatively large number of people can be affected by sudden exposure to high amounts of irradiation over a short period of time due to nuclear power plant accidents or atomic war. The extent of damage to an organism depends on the duration and the dosage of radiation with very high mortality after a threshold dose. ARS is a multi-organ failure syndrome caused by a combination of radiation dose-dependent direct cytocidal effects of irradiation on tissue stem and progenitor cells and the supporting sinusoidal endothelial and mesenchymal cells of the stem cell niche, with subsequent neutropenia, anemia, and thrombocytopenia due to bone marrow failure. With higher doses, manifestations of gastro-intestinal (GI)-ARS with loss of the intestinal mucosal barrier, bacteremia, septic shock, and systemic inflammatory response syndrome ensues. In addition to such accidental exposures, normal tissues succumb to radiation during radiotherapy in cancer patients, which is often unavoidable (Singh et al., 2018). For instance, in head and neck cancer patients, salivary glands are often present in the field of radiation (Coppes and Stokman, 2011), resulting in loss of stem cells, irreversible loss of saliva production over the years, leading to Xerostomia. Therefore, therapeutic strategies to ameliorate radiation-induced normal tissue toxicity are of great importance for tissues such as bone marrow, intestine, liver, and lung.

At the cellular level, radiation exposure inflicts direct damage by ionizing biological macromolecules such as DNA, RNA, lipids, and proteins. Indirect damage to cells occurs *via* radiation-induced generation of reactive oxygen species (ROS), such as superoxide and hydroxide radicals from the radiolysis of intracellular water, which results in the oxidation of biological macromolecules. Radiation-induced single-strand (SSBs) and double-strand DNA breaks (DSBs) are considered the major events leading to cell death, cell cycle arrest, and senescence (Panganiban et al., 2012).

Radiation medical countermeasures (MCMs) are agents administered either as preventive or as mitigators post-exposure to radiation. The mitigators improve radiation-induced physiological damage such as cellular toxicity, apoptosis, and loss of stem cells. The radiation protectants prevent radiation-induced toxicity, for instance, by scavenging the free-radicals and reducing oxidative damage to cells. Several candidate MCMs were being identified and investigated (Singh and Seed, 2020). The cytokines Neupogen® (G-CSF), Neulasta® (pegylated G-CSF), Leukine® (GM-CSF), and Nplate® (thrombopoietin receptor agonist) are radiation MCMs that received approval from the Food and Drug Administration (FDA) in the US for treating patients exposed to acute high doses of radiation that suppresses the functions of bone marrow and immune system.

### Organ Damage Induced by Radiation

**FIGURE 1:**
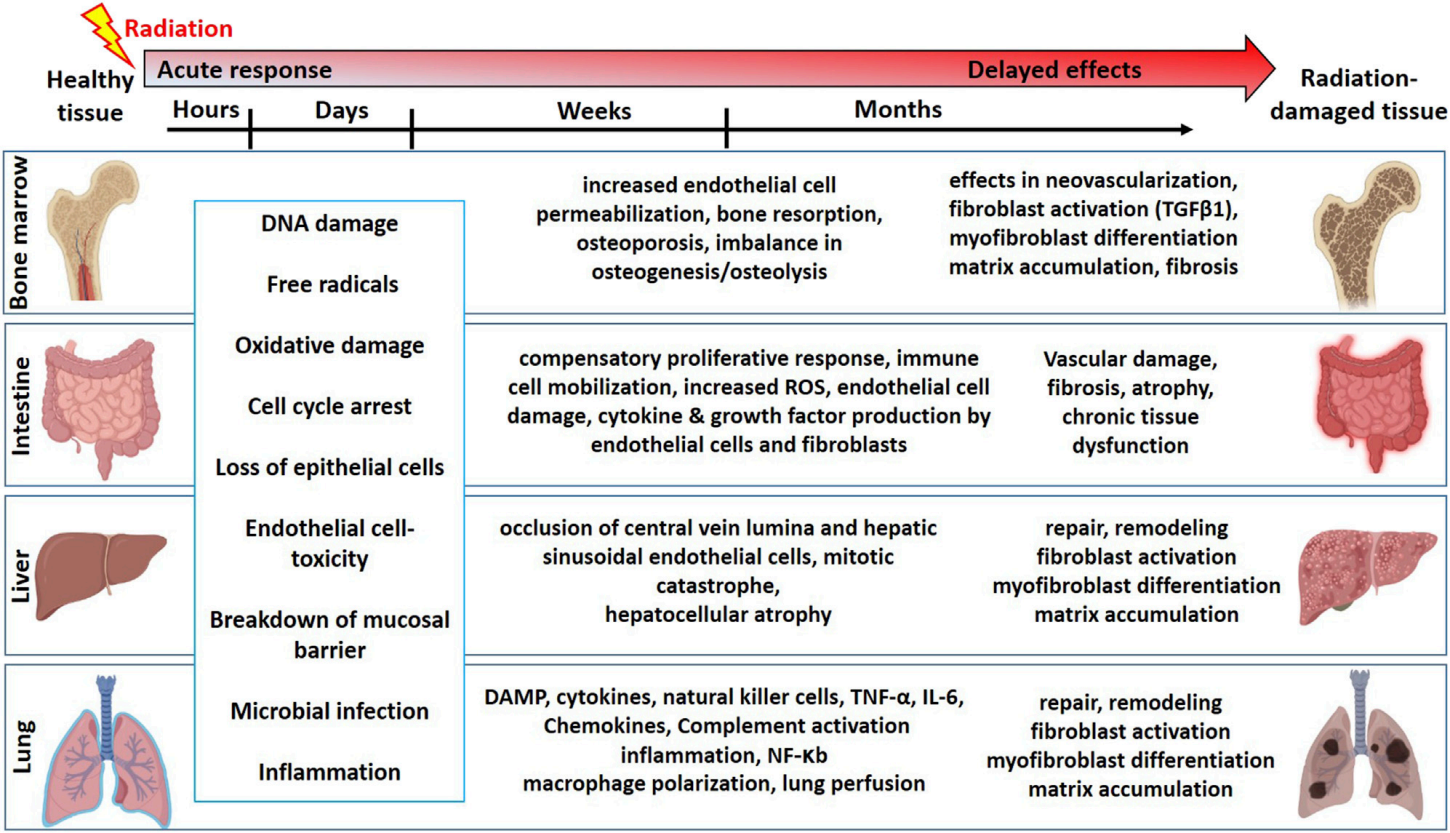
The timeline and pathophysiologic changes in the bone marrow, intestine, liver, and lung for the development of normal tissue toxicity after high dose radiation exposure (Graphics adapted from BioRender.com).

Mitotically active cells such as tissue-resident stem cells are more sensitive to radiation. Radiation-induced loss of stem cells is reported in multiple organs such as the bone marrow (Green and Rubin, 2014), intestine (Kulkarni et al., 2016), liver (Guha et al., 1999), lung (Giuranno et al., 2019), salivary gland (Coppes and Stokman, 2011; Rocchi and Emmerson, 2020), and brain (Leavitt et al., 2019). In the bone marrow, hematopoietic stem cells (HSCs) produce all the blood cell lineages (Bouchareychas et al., 2020). The HSC self-renewal capacity and differentiation potential are partly regulated by a complex multicellular network in the bone marrow microenvironment referred to as a niche. This bone marrow niche is composed of many different cell types such as mesenchymal stromal/stem cells (MSCs), adipocytes, osteocytes, and glial cells. Exposure to high-dose radiation during radiotherapy for leukemia and other bone malignancies results in the apoptosis of HSCs, decreasing their number and potential to self-renew and differentiate (Mendelson and Frenette, 2014). However, both *in vitro* and *in vivo* studies have shown that MSCs from bone marrow are relatively resistant to ionizing radiation and maintain their differentiation potential even exposure to high dose radiation (Singh et al., 2012; Nicolay et al., 2015; Ruhle et al., 2018). In addition to bone marrow cell loss, radiation also causes an increased endothelial cell (EC) permeability, imbalance in osteogenesis, damage to the bone microenvironment, and therefore infection susceptibility. The intestine is also a mitotically active tissue with actively proliferating crypt base cells identified as intestinal stem cells (ISCs), which are surrounded by MSCs, ECs, macrophages (Mɸs), and lymphocytes. In addition to accidental exposure, radiotherapy for abdominal and kidney cancer patients causes damage to the intestine, resulting in loss of intestinal crypts, loss of mucosal barrier, and leading to microbial infection and inflammation (Kulkarni et al., 2016). Lung tissue is often exposed to radiation in patients undergoing radiotherapy for lung and esophageal cancers (Giuranno et al., 2019). This results in cell loss, edema of the alveolar walls, increased vascular permeability, and inflammation. Radiation-induced damage to the healthy liver occurs during radiotherapy for hepatocellular carcinoma resulting in occlusion of central vein lumina, EC toxicity, and hepatocellular atrophy (Guha et al., 1999; Benderitter et al., 2014). In addition to these acute effects, delayed toxicities were reported in most tissues leading to multi-organ failure. A schema on summary of the timeline and pathophysiologic changes following radiation exposure in different organs is represented in Figure 1.

### Cell Therapy and Limitations

Stem cell therapy have been used to develop as radiation MCM (Rios et al., 2017). Stem cell transplantation led to the recovery of radiation-induced normal tissue toxicity in the bone (Becker et al., 1963), skin (Riccobono et al., 2012; Horton et al., 2013), salivary gland (Kojima et al., 2011; Lim et al., 2013; Nanduri et al., 2014; Maimets et al., 2016), brain (Acharya et al., 2015; Liao et al., 2017; Leavitt et al., 2019; Soria et al., 2019; Chu et al., 2020), and intestine (Saha et al., 2011; Gong et al., 2016; Zheng et al., 2016). Different types of stem cells were investigated in these studies for their potential to engraft, differentiate, repair, and regenerate radiation-damaged tissues. Allogenic bone marrow transplants are promising cell therapeutic strategies to recover radiation-induced bone marrow damage. However, challenges remain with the expansion and maintenance of HSCs *in vitro* (Walasek et al., 2012) and the ability of transplanted HSCs to engraft, self-renew and differentiate (Mendelson and Frenette, 2014). The number of resident stem cells in adult tissues is minimal and needs to be expanded *in vitro* to generate sufficient cells for the clinical translation. Moreover, stem cells' purification and selection strategies, such as flow-cytometry-based sorting, are not always suitable for clinical translation. Embryonic stem cells and genetically reprogrammed induced pluripotent stem cells (iPSC) or iPSC-derived differentiated cells such as hepatocytes, ECs are being investigated for regenerative application (Lee et al., 2019). Also, using the viral transduction methods to genetically engineer and modify the characteristics of the cells such as iPSCs poses a safety concern for clinical use. The transplantation of stem cells or MSCs may not have an immediate effect on attenuating organ injury. These transplanted cells need to be first engrafted in the body, and under some conditions, they need to further differentiate to other cell types to execute the needed biological activities. The long process of developing therapeutic effect by cell transplantation is not suitable for accidental radiation exposure where immediate repair and regenerative measures are required.

Studies have suggested that the secretome of various stem cells contains the critical growth factors and signaling molecules for the stem cell-driven regeneration *via* paracrine signaling route, mainly by extracellular vesicles (EVs) (Berger et al., 2017; Taheri et al., 2019). Therefore, EVs released from various cell types can be an alternative to cell therapeutics.

### Extracellular Vesicles Derived From Cells as Therapeutics

**FIGURE 2:**
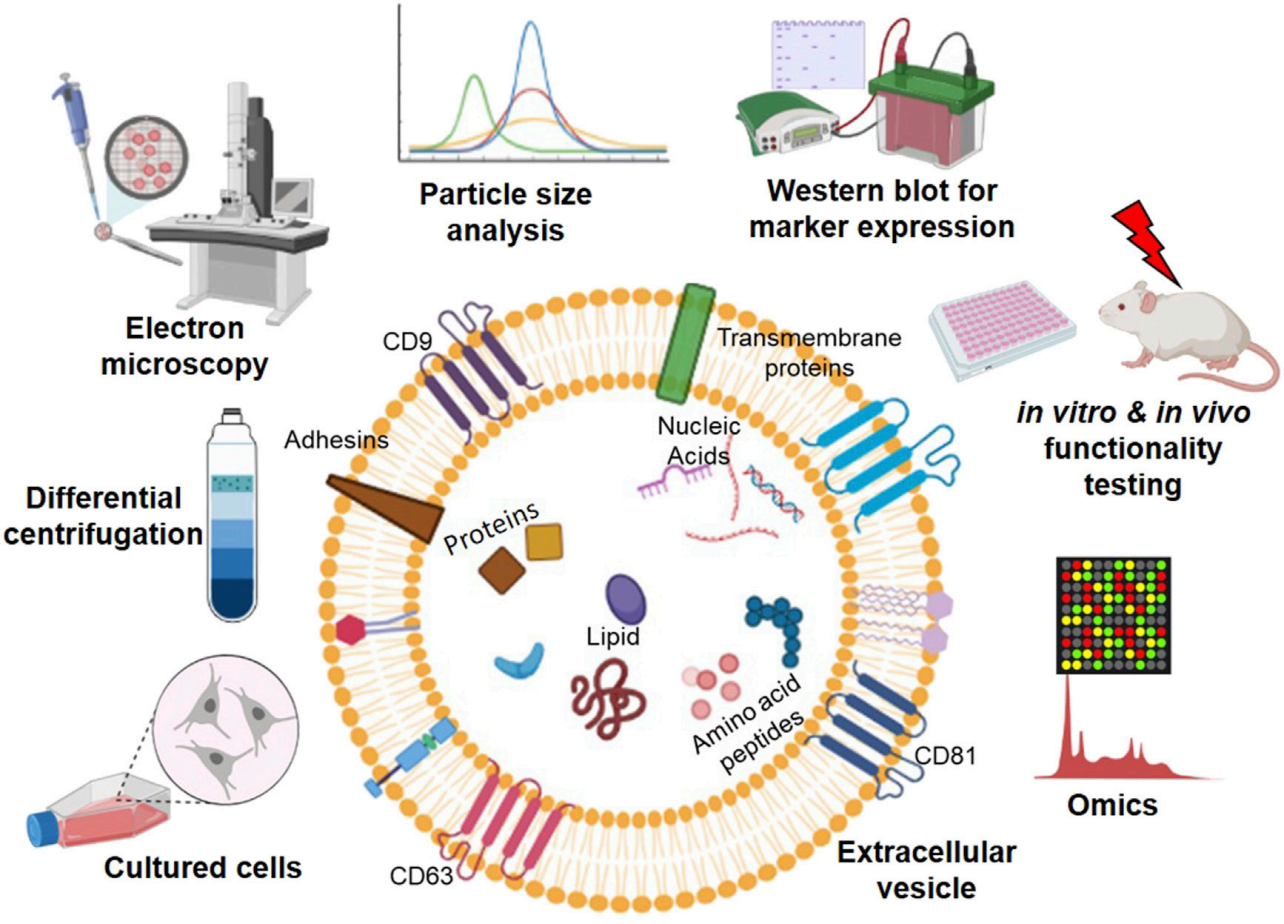
An overview of workflow in studying extracellular vesicles (EVs). EVs contain a lipid bilayer membrane that encapsulates various molecules, including proteins, nucleic acids, amino acids/peptides, and lipids, for cell-to-cell communication. EVs are secreted from the cultured cells from various sources and isolated from the cell culture medium using ultracentrifugation. The isolated EVs can be characterized by (left to right on the top of EV) electron microscopy, particle size analysis, Western blotting, *in vitro* and *in vivo* functional testing, and high throughput-omics analysis (Graphics adapted from BioRender.com).

EVs are the body's own nanoparticles that constitute a significant cell-to-cell communication system in multicellular organisms. According to the recent nomenclature stated by the International Society of Extracellular Vesicles (ISEV), EVs are broadly classified into plasma membrane-derived large microvesicles of 500–1,300 nm or endoplasmic reticulum-endosome derived small EVs of 30–200 nm (Thery et al., 2018). EV membranes contain various integrins, lipids, and proteins, each with a specific role; for instance, tetraspanins contribute to target cell selection (Rana et al., 2012). Molecules, such as CD9, CD63, CD81, tumor susceptibility gene 101 (TSG101), are used as signature biomarkers in the characterization of EVs; however, their expression levels vary, depending on the source of cell types. The content of EV and methodologies for characterizing EVs are summarized in Figure 2. Their unique nano-size, short life span makes EVs ideal messengers to travel between complex cellular fluid and selective membraned cellular structures. Depending on the cellular state of origin, EVs package either protein, small regulatory RNA, or lipids in addition to fragments of DNA (Witwer et al., 2019). However, the fate decisions that regulate this packaging are still under investigation.

Radiation stem cells and adversely affects the microenvironment. In the intestine, the stem cell niche supports ISC growth and crypt regeneration and is composed of MSCs, ECs, Mɸs, and lymphocytes which are affected due to radiation. Transplantation of mouse bone marrow adherent stromal cell (BMASC) culture system enriched for MSCs (CD90^+^/CD105^+^/CD29^+^), myeloid cells (CD45+/CD11b^+^), and ECs (CD34^+^/CD31^+^) improved the survival mice exposed to whole-body irradiation (WBI) (Saha et al., 2011). However, transplantation with the BMASC culture system depleted of myeloid cells or MSCs lost the beneficial effect. This study suggests that a combination of ECs, Mɸs, and MSCs is necessary for the complete regeneration of the damaged intestine. In this review we have focused on the bone marrow derived MSC-, EC- and Mɸ-derived EVs for radiation injuries.

## Mesenchymal Stromal/Stem Cell-EVs

Multipotent MSCs from multiple sources such as bone marrow, umbilical cord, and adipose tissue are the most widely tested stem cells. The regenerative potential of these cells is attributed to their multi-lineage differentiation potential, secretory, immune-modulatory, and homing capabilities (Viswanathan et al., 2019; Nolta et al., 2020). From 2008 to date, several clinical trials are in progress worldwide with MSCs for neurologic, cardiovascular, pulmonary, liver, bone, skin, intestinal, and muscle abnormalities, including the most recent COVID-19 (Moll et al., 2019). The most common route of administration of MSCs is intravenous; however, intraperitoneal, subcutaneous routes are also being tested clinically (Kabat et al., 2020). In pre-clinical models, MSCs were shown to be distributed to lung, heart, and kidney after systemic transplantation. Recent reports have attributed this regenerative potential of MSCs to their paracrine signaling mechanism *via* EVs (Caplan and Dennis, 2006). In general, MSC-EVs package heterogeneous cargo such as nucleic acids, protein, and lipids. Studies have shown that >150 different miRNA are present in MSC-EVs to regulate a wide range of signaling pathways (Ferguson et al., 2018). The potential of MSC-EVs to repair tissue injury was reported in the heart (Phinney and Pittenger, 2017), kidney (Zhang et al., 2016), lung (Wang et al., 2020), liver (Tan et al., 2014), intestine (Accarie et al., 2020), and cartilage (Wong et al., 2020). MSC-EVs were shown to be anti-inflammatory, anti-oxidative, pro-angiogenic (Komaki et al., 2017), anti-fibrotic (Grange et al., 2019), promote epithelial cell growth (Gatti et al., 2011; Alcayaga-Miranda et al., 2016; Tsiapalis and O'Driscoll, 2020), improve myocardial infarction (Xu R. et al., 2019), promote Mɸ M2 polarization and wound healing.

### Biological Activities of Bone Marrow MSC-EVs

Bone marrow-derived mesenchymal stromal/stem cells (BMMSCs) were first reported by Friedenstein et al. (1968). According to the International Society for Cell and Gene Therapy (ISCT), MSCs are defined as adherent cells possessing tri-lineage differentiation potential into osteocytes, chondrocytes, adipocytes, and express markers CD73, CD90, and CD105 (Dominici et al., 2006). The published materials from the last decade referred to in this review have analyzed one or a few of these properties to define the BMMSC population used to obtain EVs. Minimal information for studies of extracellular vesicles (MISEV) 2018 has given guidelines for the preparation of EVs from various BMMSCs (Thery et al., 2018; Witwer et al., 2019). Based on these guidelines, human MSCs need to be characterized as positive for CD105, CD73, CD90 expression and negative for CD45, CD34, CD14, CD11b, CD79α or CD19. In addition, the differentiation potential of human BMMSCs into osteocytes, adipocytes, and chondrocytes needs to be confirmed.

EVs isolated from mice, rats, and human BMMSCs have been studied for their regenerative potential in different organ injury models (Table 1). Mouse BMMSC-EVs were reported to promote angiogenesis *via* extracellular matrix metalloproteinase inducer (Vrijsen et al., 2016), stabilize endothelial-barrier function *via* hepatocyte growth factor (Wang et al., 2017). Rat BMMSC-EVs were reported to reduce oxidative stress *via* catalase (de Godoy et al., 2018), improve cognitive recovery, and reduce neural inflammation in the traumatic brain injury model (Zhang et al., 2015).

**TABLE 1:** The biological effect of BMMSC-EVs on organ and cell repair in various non-radiation injury models.

EV source	EV Characterization	Model	Effect	Signaling	References
Mouse BM MSCs	<200 nm	Traumatic brain injury	Cognitive recovery, neuroblast proliferation, reduced neural inflammation	-	Zhang et al. (2015)
	NTA	Necrotic entero colitis	Reduced intestinal toxicity	-	Rager et al. (2016)
Rat BMMSCs	<300 nm, EM, flow cytometry for CD81, CD63	*In vitro* model of Alzheimer's disease	Neuroprotective	Catalase, reduced oxidative stress	de Godoy et al. (2018)
	<200 nm EM, WB for HSP70, TSG101, CD63, CD81	Myocardial injury	Reduce inflammation, promote M2 macrophage polarization	NF-κB	Xu et al. (2019a)
	<200 nm, EM, WB for CD63, CD81, Alix	Myocardial infarction	Inhibition of myocardial infarction	ATG13, mTOR, autophagy	Zou et al. (2019)
	<200 nm, EM, WB for CD63, CD81, CD9	Bone fracture	Endothelial cell proliferation, osteoblast proliferation	HIF-1α-VEGF, BMP-2/Smad1/RUNX2	Zhang et al. (2020a)
Human BMMSCs	EM, immunolabeling CD9, CD63	Acute kidney injury	Proliferate proximal tubular epithelial cells	IGF-1	Tomasoni et al. (2013)
	<200 nm, EM, WB for CD9, Flotillin1	Angiogenesis assays	Endothelial cell proliferation	EMMPRIN	Vrijsen et al. (2016)
	EM, CD63 ELISA	Optical nerve crush	Retinal ganglion cell protection	-	Mead and Tomarev (2017)
	<200 nm, EM, WB for CD9, CD63, CD81, TSG101, Alix	Carbon tetrachloride-induced liver fibrosis	Improved liver function reduced inflammation and fibrosis	Wnt/β-catenin	Rong et al. (2019)
	<200 nm, EM, WB for CD9, CD63, CD81	Rat calvaria bone defect	Bone regeneration, angiogenesis	VEGF, ANG1, ANG2	Takeuchi et al. (2019)

### BMMSC-EVs for Radiation-Induced Bone Marrow Injury

Mitotically active bone marrow is highly susceptible to radiation. Exposure to radiation results in increased vascular permeability, loss of hematopoietic stem-progenitor cells, osteopenia, arrest of bone growth, and bone marrow stromal cell apoptosis, eventually leading to fibrosis (Pacheco and Stock, 2013).

Murine BMMSC (CD44^+^/CD24^+^/CD105^+^/Sca-1^+^and CD31^-^/CD11b^−^/CD45^-^/CD34^-^/CD86^-^) derived EVs could rescue bone marrow hematopoietic cells from radiation damage *in vitro* and *in vivo* (Wen et al., 2016). In this study, when treated with murine BMMSC-EVs, the irradiated mouse hematopoietic cell line (Factor Dependent Continuous-Paterson 1) showed increased proliferation and reduced apoptosis. The authors further studied the potential of mouse and human BMMSC-EVs to rescue murine hematopoietic radiation damage *in vivo*. Seven days post 1 Gy WBI of B6. SJL mice, bone marrow-lineage negative cells were isolated and cultured with 2 × 10^9^ murine MSC-EV particles/ml or vehicle for 48 h. These cells were intravenously injected into 2 Gy WBI C57BL/6 mice. Mice that received BMMSC-EV treated irradiated bone marrow hematopoietic cells showed a significant increase in engraftment at 36 weeks compared to those transplanted with vehicle-treated cells. Whole bone marrow cells that were harvested at 36-weeks post-transplant were able to recover the bone marrow after secondary transplantation into lethally irradiated mice. Similarly, intravenous injection of 4 × 10^9^ human MSC-EV particles/ml into 5 Gy WBI mice showed improved granulocytes and white blood cells at three and five weeks post-radiation.

Another report from the same group investigated the bio-distribution of DiD-labelled MSC-EVs in irradiated bone marrow using different doses, injection schedules, and timing post-radiation (Wen et al., 2019). They showed that 5 Gy WBI mice treated with BMMSC-EVs had a significant increase in the uptake of EVs by CD11b^+^ and F4/80^+^ cells in the spleen compared to that of femur bone marrow at 6 and 24 h post-radiation. Besides, an increase in uptake of EVs was observed in a radiation dose-dependent manner when injected 6 h post-radiation into mice at 1, 3, or 6 Gy radiation. This study also reported a dose-dependent increase in EV uptake in bone marrow, spleen, and liver when injected with 2 × 10^8^, 2 × 10^9^, and 2 × 10^10^ particles of BMMSC-EVs, 24 h post-5 Gy WBI. Similarly, after three intravenous injections of 2 × 10^9^ EV particles to 5 Gy WBI mice, a significant increase in EV load was observed in the liver and spleen compared to a single injection.

Zuo et al. (2019) reported that Sprague-Dawley rat BMMSC-EVs alleviate radiation-induced bone loss in rats that received 16 Gy Cesium radiation to the knee joint of the left hind limb (Zuo et al., 2019). In their study, BMMSC-EVs were isolated *via* ultracentrifugation and were <100 nm in size, expressed CD63, CD81, TSG101, and negative for Calnexin. The amount of EVs at 1.6 mg/kg or 1 × 10^6^ BMMSCs were injected into the tail vein of 16 Gy irradiated rats. Bone volume fraction (BV/TV) is the parameter to determine the volume of mineralized bone per unit volume of the sample. The BV/TV of the non-irradiated mice was 67.6%, while it was decreased to 30.9% in the irradiated mice 28 days post-radiation. However, the BV/TV of the irradiated mice transplanted with BMMSCs and BMMSC-EVs was increased by 53 and 13%, respectively, compared to the irradiated mice without transplantation. Furthermore, incubation of BMMSC-EVs with 6 Gy irradiated BMMSCs *in vitro* showed a decrease in DSB as determined by γ-H2AX staining at 2, 4, and 12 h post-radiation; an increase in antioxidant effect *via* increasing the expression of superoxide dismutase (SOD) 1 and 2 at 12 h and 24 h post-radiation and activation of Wnt/β-catenin signaling in BMMSCs, which could be the mechanisms of improving bone loss observed in irradiated rats with BMMSC-EV treatment. This study also showed that BMMSC-EVs treatment restored the radiation-induced loss of BMMSCs differentiation potential as indicated by an increase in calcium deposition, Runx2 expression (osteogenic), and oil red O staining (adipogenic) compared to irradiated cells without treatment (Zhou et al., 2018).

### BMMSC-EVs for Radiation-Induced Intestinal Injury

Stem cell-driven regeneration in the intestine is mediated by cycling leucine-rich repeat-containing G-protein coupled receptor 5 (Lgr5^+^) stem cells at the crypt base *via* a Wnt signaling pathway (Barker et al., 2007). These crypts are in close contact with stromal cells such as mesenchymal cells, ECs, Mɸs, and lymphocytes that provide the signaling factors for intestinal regeneration. Radiation damage to the intestine leads to the loss of these rapidly cycling Lgr5^+^ stem cells to the impairment of epithelial regeneration, showing an irreversible loss of crypt-villi, EC apoptosis, and loss of mucosal barrier. Collectively these contribute to septic shock and systemic inflammatory response as a radiation-induced gastrointestinal syndrome.

Accarie et al. (2020) has shown that EVs that are <250 nm and CD81^+^ derived from human BMMSCs mitigate intestinal toxicity in a mouse model of ARS (Accarie et al., 2020). This study compared the effect of intravenous injection of 600 µg of BMMSC-EVs after 10 Gy WBI. A 3.5-days delay in death was observed in nude mice that received three injections of BMMSC-EVs at 6, 24, and 48 h post-10 Gy WBI in comparison to untreated, irradiated control mice. Also, the expression of tight junction protein claudin-3 was more preserved at the membrane of small intestine epithelium in BMMSC-EV treated mice than in irradiated, non-treated controls. At 3 days post-WBI, a dose-dependent decrease in apoptotic cells, an increase in Ki67^+^ cells in the crypts, and less alteration of crypt-villus architecture was observed in BMMSC-EV-treated mice in comparison to the irradiated mice without treatment.

The direct effect of human BMMSC-EVs on the ISCs after radiation has not been studied yet. Reserve ISCs like radio-resistant cells expressing Keratin19 (Krt19) (Asfaha et al., 2015) and Polycomb complex protein (Bmi1) (Yan et al., 2012) were shown to be generating Lgr5^+^ cells for recovering the functional cell loss after radiation. However, other growth factors and EVs from multiple cell sources that can stimulate these stem cells need to be further investigated.

### BMMSC-EVs for Radiation-Induced Liver Injury

During radiotherapy for hepatocellular carcinoma, normal liver tissue is often exposed to radiation. This results in liver sinusoidal endothelial cell toxicity, atrophy of hepatocytes, and occlusion of veins, gradually leading to loss of liver function and progressing toward radiation-induced liver disease (RILD) or radiation hepatitis (Benderitter et al., 2014). An increase in inflammatory cytokines such as tumor necrosis factor-alpha (TNF-α), interleukin (IL)-1β, and IL-6 was observed in the early phase of RILD. Currently, there is no effective treatment for RILD.

Congenic hepatocyte transplantation *via* intra-splenic injection is shown to repair acute and late effects of RILD (Guha et al., 1999). Intra-splenic transplantation of liver sinusoidal endothelial cells combined with hepatocyte growth factor into partial hepatic irradiation rodent model was shown to ameliorate radiation-induced sinusoidal obstructive syndrome and repopulate the irradiated sinusoidal endothelium by eight weeks after transplantation (Kabarriti et al., 2010). Challenges with orthotropic liver transplantation and the minimal availability of donor hepatocytes for safe transplant limit the use of hepatocyte transplantation to treat liver diseases.

EVs derived from MSCs from the human umbilical cord (Li et al., 2013), bone marrow (Rong et al., 2019), and embryonic stem cells (Tan et al., 2014) have been reported to alleviate liver injury in drug-induced hepatic injury models (Lou et al., 2017; Phinney and Pittenger, 2017). Herrera et al. (2010) have shown that microvesicles derived from human liver stem cells (CD29^+^/CD44^+^/CD73^+^/CD90^+^) accelerated liver regeneration in 70% hepatectomized rats (Herrera et al., 2010). Rong et al. (2019) showed that human BMMSC-EVs (<200 nm) effectively alleviated liver fibrosis by inhibiting Wnt/β-catenin signaling in the carbon tetrachloride -induced liver damage model (Rong et al., 2019). Even though MSC-EVs were shown to alleviate various drug-induced or physical hepatic injury models, their potential to recover radiation-induced liver injury is not well studied.

### BMMSC-EVs for Radiation-Induced Lung Injury

Radiation-induced pneumonitis and radiation-induced pulmonary fibrosis are the major early and delayed lung toxicities in cancer patients undergoing thoracic radiotherapy. Radiation-induced DNA damage and free radicals result in epithelial cell death leading to lung mucositis. This is followed by an increase in inflammatory cytokines (TNF-α and IL-6), natural killer (NK) cells, Mɸ polarization, edema, and lung perfusion, eventually activating fibroblasts and myofibroblast differentiation to fibrosis in the irradiated lung (Kabarriti et al., 2020). MSCs derived from the umbilical cord (Wei et al., 2020), bone marrow (Lee et al., 2012), and adipose tissue (Dong et al., 2015) were reported to be attenuating various models of lung injury.

Human umbilical cord-MSCs could repair radiation-induced lung injury (RILI) by inhibiting myofibroblastic differentiation of human lung fibroblasts (Xu S. et al., 2019; Zhang et al., 2019). Human adipose-MSCs were shown to downregulate TNF-α signaling in the 15 Gy-irradiated lungs and prevent the epithelial-mesenchymal transition of irradiated type II alveolar epithelial cells (Dong et al., 2015). Klein 2017 showed that conditioned media from aorta-MSCs restored SOD1 expression and protected EC loss in the lung of RILI mice (Klein et al., 2017). These studies have demonstrated the effectiveness of using different sources of MSCs, which warrants further investigation on MSC-EVs for treating RILI.

## Endothelial Cell-EVs

The heart pumps the blood carrying nutrients and oxygen to all the cells in our body *via* arteries to capillaries and collects the blood *via* veins for waste removal and purification. The collective action of the blood vascular system and lymphatics maintains the fluid level in the body. All these vessels are lined by ECs that play an important role in vascular homeostasis. In addition, ECs mediate immune responses, involve in inflammation, coagulation, and angiogenesis (Orfanos et al., 2004). ECs differ based on their location; for instance, micro versus macrovascular ECs have a distinct response to physiologic and inflammatory stimuli (Stevens et al., 2008).

Radiation induces EC dysfunction, such as increased permeability, apoptosis, and detachment from the basement membrane of the vessels (Flamant and Tamarat, 2016). This often leads to inflammation, fibrosis, and damage to tissue microvasculature depending upon the radiation dose. Radiation-induced EC toxicity is reported in various tissues such as the intestine (Paris et al., 2001; Wang et al., 2007), lungs (Ghobadi et al., 2012; Ziegler et al., 2017), central nervous system (Peña et al., 2000), and parotid glands (Xu et al., 2010). Ionizing radiation-induced long-term senescence was reported in ECs (Lafargue et al., 2017). Prevention or inhibition of EC toxicity was reported to protect the intestine (Rotolo et al., 2012), central nervous system (Peña et al., 2000), and lungs (Klein et al., 2017) against radiation-induced damage. EC transplantation has been shown to be beneficial in mouse models of hemophilia (Follenzi et al., 2008), hepatectomy (Melgar-Lesmes et al., 2017), and lethal irradiation in mice (Chute et al., 2007).

### Biological Activities of EC-EVs

Like many mammalian cells, ECs respond to stimuli and produce heterogeneous cargo containing EVs. A distinct proteomic cargo was reported in human umbilical vein endothelial cells (HUVECs) when stimulated with TNF-α (Letsiou and Bauer, 2018). Similar changes were reported in the proteome cargo of EVs from human pulmonary artery endothelial cells treated with mechanical cyclic stretch or lipopolysaccharides (Letsiou et al., 2015). Van Balkom 2015 analyzed the miRNA profile of human microvascular endothelial cells (HMEC-1) and thus-derived EVs. The study showed that EC-EVs contain miRNA’s related to the regulation of angiogenesis, proliferation, and differentiation (van Balkom et al., 2015). Endothelial progenitor cell-EVs were shown to improve atherosclerotic endothelial dysfunction in a mouse model of atherosclerotic diabetes (Bai et al., 2020). EC-EVs were shown to enhance EC proliferation (Wang et al., 2019) and act against apoptosis and inflammation (Andrews and Rizzo, 2016). EC-EVs from various sources were shown to be neuroprotective (Xiao et al., 2017), improve sepsis (Zhou et al., 2018), and improve high D-glucose-induced endothelial dysfunction (Saez et al., 2018).

HUVEC-EVs were shown to protect human neuroblastoma SH-SY5Y cells from ischemia-reperfusion injury *in vitro* (Xiao et al., 2017). Endothelial colony-forming cells (ECFC)-EVs inhibited apoptosis and reduced ischemic kidney injury in mice (Vinas et al., 2018). Brain EC-EVs promoted motor function and Synapsin I expression in the cerebral artery occlusion model of rats (Gao et al., 2020). EVs from HUVECs cultured in high glucose media restored wound healing of basal glucose cultured HUVECs compared to EVs from HUVECs cultured in basal media (Saez et al., 2018). EVs isolated from HUVECs cultured under high glucose increase intercellular adhesion molecule 1 (ICAM1) expression in Mono-Mac-6 cells, a monocytic cell line (Saez et al., 2019). HUVEC-EVs showed a decrease in cardiomyocyte death, protected against hypoxia *via* extracellular signal-regulated protein kinase (ERK1/2) and mitogen-activated protein kinase (MAPK) signaling (Davidson et al., 2018). The cardioprotective effect was not observed when treated with EV depleted conditioned media. Increased axonal growth and upregulation of miRNA related to the regulation of Sema6A, Phosphatase and Tensin Homolog (PTEN), and RhoA was observed with rat cerebral EC-EVs *in vitro* (Zhang Y. et al., 2020). Vascular smooth muscle cells showed an increased vascular cell adhesion molecule 1 (VCAM1) expression and leukocyte adhesion when cultured with rat cerebral EC- EVs (Boyer et al., 2020). The summary of utilizing various biological activities of EC-EVs in treating different non-radiation injury models discussed in this review are listed in Table 2.

**TABLE 2:** The biological effect of endothelial cell-EVs on organ and cell repair in various non-radiation injury models.

EV source	EV Characterization	Model	Effect	Signaling	References
HUVECs	<200 nm, EM, WB for CD9, HSP70, TSG101	Cerebral ischemia-reperfusion injury	SH-SY5Y nerve cell protection	-	Xiao et al. (2017)
ECFCs	<200 nm, WB for CD81, TSG101	Kidney ischemic injury	Inhibition of apoptosis, reduced ischemic injury	miR-486–5p, PTEN	Vinas et al. (2018)
Senescent HUVECs	EM, WB for CD63, CD9; Calnexin, β-actin negative	HUVECs *in vitro*	Decreases in VE-cadherin, β-catenin, decreased cell growth and impaired migration potential	β-catenin	Wong et al. (2019)
HUVECs	<200 nm, EM, flow cytometry for CD63	Adult rat cardiomyocytes co-culture *in vitro*	Decreased cell death of cardiomyocytes, protection against hypoxia	ERK1/2, MAPK	Davidson et al. (2018)
HUVECs conditioned with basal and high glucose	<300 nm, EM, WB for CD63, CD81	HUVECs growth, wound healing *in vitro*	Induced endothelial dysfunction in HUVECs	ICAM-1	Saez et al. (2018)
HUVECs and monocytes	<300 nm, EM, WB for CD63	Monocytes (MM6) and HUVECs under high glucose *in vitro*	Increase ICAM-1 expression in MM6 cells	ICAM-1	Saez et al. (2019)
Brain ECs (bEnd.3)	EM	Rat cerebral artery occlusion model	Promoted motor function, synapsing in dendrites	miR-126–3p	Gao et al. (2020)
Rat cerebral ECs (CECs) and ischemic-CECs	<200 nm, EM, WB for CD63, CD31, Alix; calnexin, zo-1 negative	Axon culture *in vitro*	Increased axonal growth, upregulation of miRNA	Sema6A, PTEN, and RhoA	Zhang et al. (2020b)
Rat aortic endothelial and vascular smooth muscle cells	<200 nm, EM, WB forTSG101, Flotillin; VDAC negative	Vascular smooth muscle cells *in vitro*	Increased VCAM1 expression and leukocyte adhesion to vascular smooth muscle cells	HMGB1	Boyer et al. (2020)

### EC-EVs for Radiation-Induced Bone Marrow Injury

The bone marrow microenvironment regulates HSC fate in homeostasis and after injury (Mendelson and Frenette, 2014). In addition to osteoblasts and stromal cells, ECs occupy a significant role in niche signals for HSCs, and EC toxicity is one of the major consequences of multi-organ failure in ARS. Piryani et al. (2019) studied whether EC-EVs could regulate HSC regeneration after ionizing radiation (Piryani et al., 2019). EVs were isolated from bone marrow ECs of C57BL/6 mice *via* differential centrifugation to obtain <200 nm in size and expressed CD31, vascular endothelial (VE)-cadherin. At 24 h after either 5 Gy (hematopoietic assays) or 8 Gy (for survival) WBI, 1.9 × 10^9^ particles of EV were intravenously injected daily for four days. Irradiated mice with EC-EV treatment showed improved bone marrow cellularity, hematopoietic stem and progenitor cell content, preserved EC architecture, and showed a 50% survival advantage. Among the 48 cytokines tested, EC-EVs increased the expression level of tissue inhibitor of metalloproteinase 1, which is essential in vascular remodeling post-ischemia (Mandel et al., 2017). The above study suggests that EC-EVs have the potential to protect post-radiation damaged ECs.

A thorough investigation needs to be performed to examine EC-EVs as radiation MCM to mitigate injury in other organs such as the intestine, liver, and lung. Specific types of ECs have distinct potential and actions (Rafii et al., 2016). The characteristics of EVs from various cell types such as the aorta, endothelial progenitor cells, and organs such as lung and liver need to be studied to facilitate tissue-specific regeneration after radiation injury.

## Macrophage-EVs

Macrophages are the phagocytic immune cells that originate from monocytes and circulate in the blood. They differentiate in various tissues as tissue-resident Mɸs such as alveolar Mɸs in the lung, Kupffer cells in the liver, microglia in the brain, and splenic Mɸs in the spleen. They are distinguished by their morphology, the pathogens they interact with, the levels and type of cytokines they produce. They present antigens to T-cells and initiate inflammation, release cytokines that activate other immune cells. Inflammatory monocytes and tissue-resident Mɸs play a crucial role in tissue repair, regeneration, and fibrosis. Insults to healthy tissues result in increased release of damage-associated molecular patterns (DAMPs). This initiates an inflammatory cascade involving recruitment, proliferation, and activation of various hematopoietic and non-hematopoietic mediators (neutrophils, Mɸs, innate lymphoid cells (ILCs), NK cells, B cells, T cells, fibroblasts, epithelial cells, ECs, and stem cells) which collectively work for tissue repair (Wynn et al., 2013). Mɸs are a great source of Wnt ligands that activate epithelial regeneration potential in injury models of the liver (Boulter et al., 2013) and kidney (Lin et al., 2010). However, Mɸs undergo reprogramming in response to the damage signals and can be pro-inflammatory M1 or anti-inflammatory M2 phenotype. M1 and M2 Mɸs possess distinct chemokine profiles and differ in the metabolism of iron, folate, and glucose (Mantovani et al., 2013). Mɸs are studied widely for their role in tissue repair and their potential for tissue regeneration.

### Biological Activities of Mɸ-EVs

Mɸ-EVs are gaining a lot of interest as therapeutics. Tissue repair potential of Mɸ-EVs was reported in models of atherosclerosis (Bouchareychas et al., 2020), cardiac injury (Dai et al., 2020), wound healing (Li et al., 2019), hair loss (Rajendran et al., 2020), dextran sulfate sodium induced-colitis (Schubart et al., 2019; Yang et al., 2019), vascular repair in intravascular stunt-implant (Wallis et al., 2020) and skin diseases (Kim et al., 2019). Mɸ-EVs from various sources were shown to be angiogenic (Yan et al., 2020), influence neural action potential (Vakili et al., 2020), anti-inflammatory, and could convert M1 to M2 polarization (Kim et al., 2019). M2 Mɸ-EVs were shown to be anti-inflammatory in the atherosclerosis model by reducing nuclear factor kappa-light-chain-enhancer of activated B cells (NF-κB) and TNF-α signaling (Bouchareychas et al., 2020). Mɸ-EVs were shown to promote Wnt-signaling for hair growth (Rajendran et al., 2020) and intestinal regeneration (Saha et al., 2016). The summary of various biological activities of Mɸ-EVs in treating different non-radiation organ injury models as discussed in this review is listed in Table 3.

**TABLE 3:** The biological effect of macrophage-EVs on organ repair in various non-radiation injury models.

EV source	EV Characterization	Model	Effect	Signaling	References
Murine bone marrow-derived macrophages (BMDM) and BMDM-treated with IL-4	<200 nm, EM, WB for CD9, Alix, Flotillin	Atherosclerosis	Reduced excessive hematopoiesis in bone marrow, number of macrophages; reduction in necrotic lesions	miRNA regulation of NF-kB, TNF-a	Bouchareychas et al. (2020)
Murine RAW 264.7 cells	<200 nm, EM, WB for CD63, Alix	Diabetic rat	Inhibited secretion of pro-inflammatory cytokines, induced endothelial cell proliferation, migration and re-epithelialization of the wound	TNF-α, IL-6 inhibition, P-AKT activation	Li et al. (2019)
Murine bone marrow-derived M2b macrophages	<200 nm, EM, WB for CD9, CD63, CD81	DSS-colitis	Increase in Treg cells, IL-4 in the spleen, suppression of IL-1β, IL-6, IL-17A	CCL1/CCR8	Yang et al. (2019)
Murine bone marrow-derived M2 macrophages	<200 nm, EM, WB for CD63, Alix	Cutaneous wound mice model	Increased M2 at the wound site, increased angiogenesis, re-epithelialization and collagen deposition	Activation of arginase, inhibition of iNOS	Kim et al. (2019)

### Mɸ-EVs for Radiation-Induced Intestine Injury

Saha et al. (2011) have developed an enrichment culture system from mouse BMASCs containing MSCs (CD90^+^/CD105^+^/CD29^+^), myeloid cells (CD45+/CD11b^+^), and ECs (CD34^+^/CD31^+^) (Saha et al., 2011). After transplanting into mice subjected to 18 Gy abdominal radiation, BMASCs have promoted ISC regeneration and improved their survival, whereas either depletion of myeloid cells or MSCs failed to regenerate the irradiated intestine, suggesting that myeloid cells and MSC mediated regeneration. Mɸs support crypt regeneration, coordinate signals from gut microbes, injured epithelium, and help ISC regeneration (Pull et al., 2005). Mɸs activated by toll-like receptor 9 was shown to ameliorate intestinal injury post-radiation (Saha et al., 2011). Wnt signaling has an important role in ISC proliferation and regeneration in the intestine. Earlier reports from our group by Saha et al. (2016) have studied Mɸ-EVs for intestinal regeneration. To understand the specific role of Wnt signing from Mɸs for intestinal repair, this study generated mice with macrophage-restricted ablation of Porcupine (*Porcn* ^fl/fl^), a gene essential for Wnt synthesis. These *Porcn-*depleted (null) mice have normal intestine but are hypersensitive to radiation injury compared to wild-type mice. The intestine in these mice showed loss of Lgr5+ cells, reduced crypt depth and number, and decreased survival after WBI (Saha et al., 2016). These mice were rescued from radiation lethality when treated with conditioned medium from wild-type bone marrow macrophages (BMM) but not with medium from Porcn-null mice BMM.

Furthermore, they have isolated EVs from the BMM-conditioned media using ultracentrifugation. These EVs were positive for TSG101, CD9, and Alix. When tested using T-cell factor/lymphoid enhancer factor (TCF/LEF)-luciferase reporter cell line, BMM-EVs showed activation of Wnt signaling. BMM-conditioned media improved the survival of mice post-18.5 Gy abdominal radiation. In contrast, the EV depleted media could not rescue, indicating that BMM-EVs carrying Wnt ligands that improve intestinal regeneration after radiation injury.

## EVs for Radiation MCM

**FIGURE 3:**
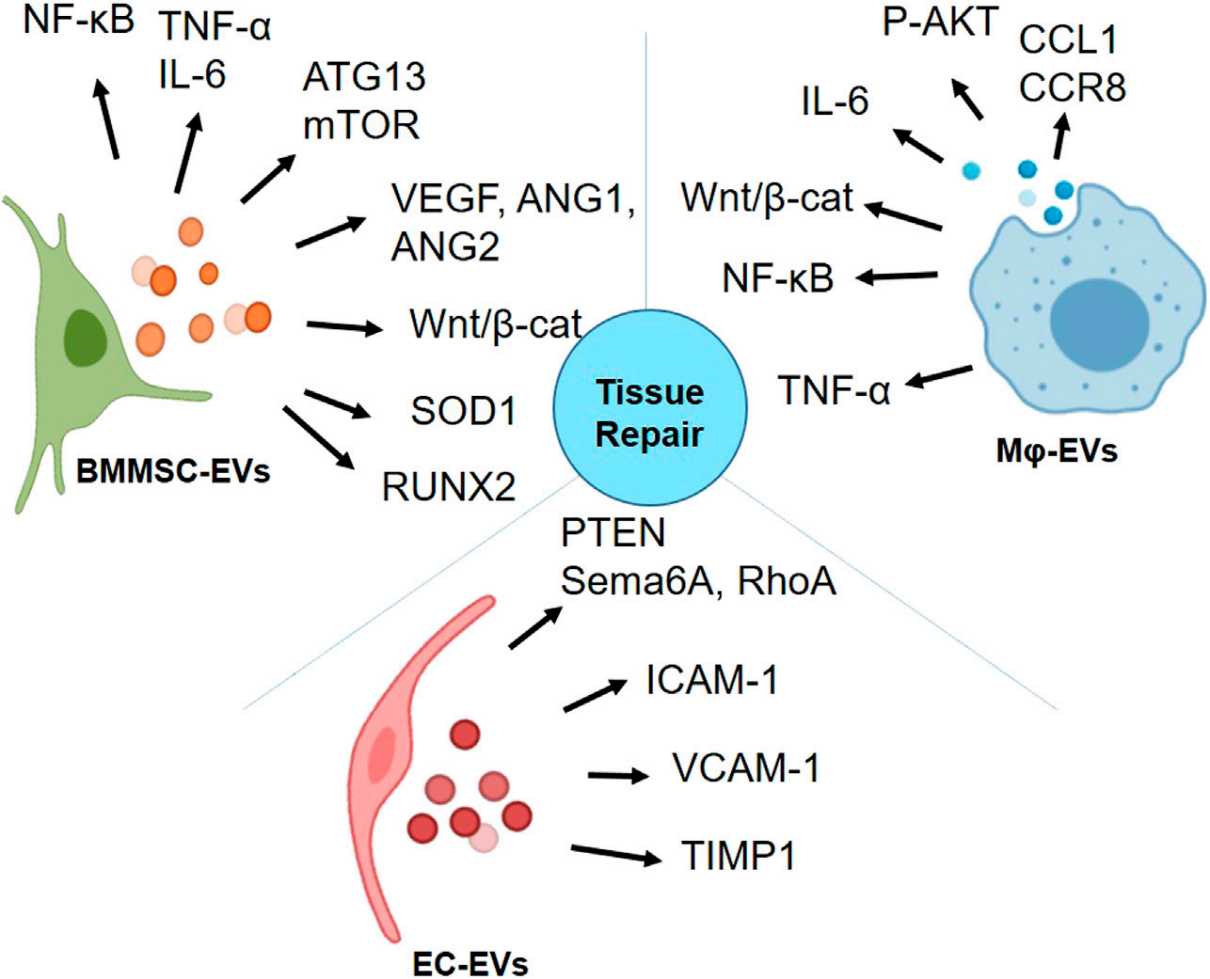
Activation of proteins involved in various signaling pathways by bone marrow mesenchymal stromal/stem cell (BMMSC)-extracellular vesicles (EVs), endothelial cell (EC)-EVs, and macrophage (Mɸ)-EVs for tissue repair (Graphics adapted from BioRender.com).

The potent anti-inflammatory, anti-fibrotic characteristics of BMMSC-EVs, angiogenic and anti-inflammatory properties of EC- and Mɸ-EVs make them promising candidates for regenerative application. The summary of EVs derived from these three cell types that have been discussed in this review on interacting with various signaling molecules is depicted in Figure 3. With such diverse potential, EVs from these cells have been extensively studied for radiation-induced injury. In Table 4, we summarized the potential of BMMSC-, EC- and Mɸ-EVs in repairing bone marrow, intestine, and lung as discussed in this review.

**TABLE 4:** Application of BMMSC-, EC- and macrophage-EVs for organ repair in radiation injury models.

Stromal Cell Source	Characteriz-ation	EV Isolation method	EV Characterization	Target tissue	Model tested	Route of administr-ation	Dose of EV	Storage	References
Human MSC (Lonza, MD, USA #PT-3001)	NA	NA	Average of 231.3 ± 124.6 nm, EM, WB for CD9, CD63, CD81, TSG101, HSP70	Bone marrow	5 Gy WBI in C57BL/6 mice	Intravenous	2 × 10^8^, 2 × 10^9^ and 2 × 10^10^ particles/mouse	PBS with 1% DMSO, at -80°C	Wen et al. (2019)
Rat bone marrow	Negative for CD34, CD45; Positive for CD29, CD44 and CD90	Differential centrifugation	EM, WB CD63, CD81; Negative for Calnexin	Bone marrow	16 Gy Knee joint irradiated Sprague-Dawley Rats	Intravenous	1.6 mg/kg	PBS, at -80°C	Zuo et al. (2019)
Murine and human bone marrow	Negative for CD31, CD45, CD11b, CD34 and CD86; Positive for CD44, CD29, CD105, Sca-1	Differential centrifugation	NanosightNS500, EM, WB for CD9, CD63, CD81	Bone marrow	2, 5 and 9.5 Gy Cesium WBI	Intravenous	4 × 10^9^ particles/mouse	10% DMSO, at -80°C for 6 months	Wen et al. (2016)
Immortalized E1-MYC 16.3 human embryonic stem cells	Negative for CD45; Positive for CD73, CD105	Tangential flow filtration	<200 nm, EM	Intestine	WBI in nude mice	Intra-venous	600 µg of EV, 6h, 24h, and 48h post-WBI	Paracrine Therapeutics's-Proprietary technique, stored at -20°C	Accarie et al. (2020)
Mouse bone marrow endothelial cells	NA	Differential centrifugation	<200 nm; WB for CD31, VE-cadherin; EM	Bone marrow	5 and 8 Gy Cesium WBI	four days i.v., starting 24 h post-WBI	1.9 × 10^9^ particles of EV per injection	NA	Piryani et al. (2019)
Mouse bone marrow macrophages	Positive for CD11b	Differential centrifugation	WB for CD9, TSG101, Alix	Intestine	18.5 Gy Abdominal Irradiation	Intra-venous	500 µl of EV and conditioned media	NA	Saha et al. (2016)

The success of radiation MCM depends mainly on the efficacy, timing, and dosage of treatment into radiation-damaged tissues. The bio-distribution of EVs *in vivo* is the crucial driver in the success of MCM and is influenced by the damage model, cell source of EVs, and route of administration. The route of administration of EVs affects biodistribution to various organs (Wiklander et al., 2015). Studies have shown that modification of EVs will influence their biodistribution *in vivo* (Royo et al., 2020). Therefore, strategies to modify EVs for targeted organ-specific delivery are of great interest for radiation injuries. For example, tissue-specific ECs were known to have specific functions (Rafii et al., 2016). This can be further investigated in terms of EVs from tissue-specific ECs. For instance, lung EC- EVs preferentially distribute to the lung over other tissues providing a lung-targeted therapy.

Alternately, cutting-edge gene-editing technology can be applied to improve or modify the characteristics of EVs. For example, EVs derived from engineered cells overexpressing key growth factors such as Wnt ligands, epidermal growth factor, and fibroblast growth factor could be tested in radiation injury models. Though the overall effect of EVs in radiation-injury models was published, the detailed molecular mechanism of EVs for radiation repair is still not well understood.

### EVs for ARS

Whole-body exposure to high doses of radiation in a short duration leads to the development of ARS, often characterized by damage to hematopoietic, gastrointestinal, and neurovascular systems. It is implacable to conduct a human clinical trial to test radiation MCMs. Instead, FDA has provided “Animal Rule” as guidance for radiation MCM approval. Animal models of the whole body, partial body, or abdominal radiation are often used to develop radiation MCMs for ARS. For treating the acute effects of radiation during the accidental event, the therapeutic agents may not be accessible immediately. Therefore, evaluating the efficacy of the radiation MCM candidates is set to be administered at least 24 h after radiation exposure for the first dose. EVs are suitable to develop as radiation MCMs due to their targeted action in a very short duration. However, multiple doses of EVs might be necessary to enhance their efficacy. On the other hand, radiation damage like ARS involves multi-organ failure, and single cell-source derived EVs with specific signaling might not yield a successful recovery. Therefore, a combination of the stem, immune, differentiated, or reprogrammed cell-derived EVs might be necessary for a broader range of efficacy.

### EVs for Delayed Effects of Acute Radiation Exposure

In addition to the acute effects, delayed effects of acute radiation exposure (DEARE) are reported in ARS survivors. Irradiation injury causes DNA damage through free radicals, SSBs, and DSBs. Even though the DNA damage is repaired, the process is not always efficient. The defects in the DNA damage response pathway result in the development of either cell cycle arrest (mitotic catastrophe, senescence) (Li et al., 2018) or cell death (apoptosis, necrosis, and autophagy). Cells in the senescence stage undergo mitotic arrest while being metabolically active. Senescent cells can cause chronic inflammation and disruption of surrounding tissue structure and function *via* the production of ROS, inflammatory mediators (IL-6, IL-18, and TGF-β), growth factors, and extracellular proteases. This process collectively refers to as senescence-associate secretory phenotype (SASP) (Li et al., 2018). The molecules secreted from SASP can also be packed in EV format (Misawa et al., 2020). This persistent insult from SASP leads to the delayed effect of irradiation-induced senescence, such as fibrosis in the lung (Citrin et al., 2013; He et al., 2019), oral mucositis (Iglesias-Bartolome et al., 2012), cardiovascular disease (Stewart et al., 2013), hypo-salivation (Peng et al., 2020), hematopoietic cell senescence (Wang et al., 2011). It has also been shown that low radiation can affect the autophagic flux, and activation of autophagy may decrease the senescence induced by radiation and prevent deterioration (Alessio et al., 2015).

Further SASP can lead to radiation-induced bystander effect (RIBE) (Uribe-Etxebarria et al., 2017). RIBE is a condition in which the radiated cells cause a stress response in the neighboring cells resulting in DNA damage, apoptosis, genomic instability, and cell death. Previously cell-to-cell communication of various components of SASP such as IL-6, IL-8, and TGF-β were considered as inducers of senescence in neighboring cells. However, the newly emerging focus is that EVs secreted from irradiated cells also played an essential role in modulating senescence in non-irradiated surrounding cells. For example, non-irradiated cells became senescent when treated with EVs isolated from irradiated cells. This observation was made in primary human fibroblasts isolated from neonatal foreskin (Elbakrawy et al., 2020), breast epithelial cancer cells (Al-Mayah et al., 2015), salivary gland stem/progenitor cells (Peng et al., 2020).

In an *in vivo* study, the damaged DNA foci were significantly higher in non-irradiated mouse fibroblast cells when treated with the irradiated mouse's serum-EVs than non-irradiated mouse serum-EVs (Ariyoshi et al., 2019). In another study, downregulation of antioxidant enzyme genes and cellular redox system (iNOS2) genes was observed in non-irradiated mice as a bystander effect when injected with bone marrow-EVs from irradiated mice (Hargitai et al., 2021). In the same token, recent evidence suggests that EVs as SASP, secreted from senescent cells might contribute to tumorigenesis and age-associated pathologies (Misawa et al., 2020). It can be a potential mechanism to explain a high incidence of cancer developed in the survivors after radiation exposure.

Senescent cells can be targeted for therapeutic purposes, i.e., *via* senolytic drugs. These drugs are usually small molecules that can selectively remove senescent cells. In comparison, senolytics can be used as MCMs for DEARE such as fibrosis.

The research for developing MSCs or MSC-EVs as senolytics is limited. MSC therapy alleviates irradiation-induced bronchial–alveolar epithelial cellular senescence and inhibits the secretion of SASP factors such as the chemokine (C-C motif) ligand 2 (CCL2) and urokinase-type plasminogen activator (Plau/uPA). In this study, aortic MSC and BMMSCs were administered at 0.5 million cells 24 h after irradiation (Klein et al., 2016). Similarly, EVs from the human placenta MSC reduced the senescence in the ECs after whole thoracic radiation. The miRNA-214-3p from EVs inhibited the Ataxia telangiectasia mutated signaling pathway of senescence in ECs and further downregulated the expression of SASP factors, resulting in attenuation of fibrosis (Lei et al., 2020). Therefore, MSC-EVs have the potential to act as senolytics, further reducing the DEARE injury in normal cells.

## Challenges in Developing EVs for Clinical Use

### Characterization and Production

EVs are classified based on immunolabeling of EV surface proteins such as tetraspanins, integrins, cell adhesion molecules, and growth factor receptors (Lotvall et al., 2014; Buzas et al., 2018). The heterogeneity in size and content of the EV populations makes it challenging to purify a single EV population (Kowal et al., 2016). Therefore, the basic understanding of the size, either with dynamic light scattering-based techniques or electron microscopy; immunolabeling with markers such as CD63 and CD9 are necessary for the characterization of the EV population of interest. EVs produced by even a single cell type have subpopulations within; therefore, more numbers of surface markers need to be analyzed. An antibody array such as Exo-CheckTM (System Biosciences) measures up to 8 surface markers expressed on EVs in a single sample is advantageous. Using immortalized stable cell lines as a source of EVs can be a way to obtain pure, sub-population-specific EVs. Alternately, methods that can design and control the cargo in EVs, such as genetically engineered cell lines, will promote EVs as drug delivery systems with a targeted action (Luan et al., 2017).

Studies that report specific cell-derived EVs should include the passage number of cells, seeding density, and culture conditions, which are essential parameters affecting the production and characteristics of EVs. Also, the dose of EVs used in pre-clinical models is often represented as micrograms of protein or particle number in some studies. Though particle concentration seems to be an ideal reference, such state-of-the-art facilities measuring particle number and concentration of EVs are not always available in every research laboratory. The number of EVs obtained from 1 million mouse BMMSCs under specific culture conditions might differ among each laboratory. Therefore, additional information as mentioned in Tables 1–4 will help researchers compare their results. Also, it will expand our knowledge on the average EV production potential of different cell types under specific culture conditions.

EVs derived from specific cell types interact with distinct signaling molecules, as described in Figure 3. Therefore, knowledge of various signaling pathways that specific cell-EVs can modulate is a prerequisite for developing targeted therapies. Luciferase reporter constructs containing specific promoter response elements are used to generate stable cell lines to monitor the activity of the transcription factors. Upon addition of specific ligands or test compounds with predicted activation, reporter activity can be measured by adding a light-emitting-substrate for the quantification. For example, HEK293 cells having TCF/LEF luciferase reporter construct have been used to monitor the Wnt activation by mouse BMM-EVs (McBride et al., 2017). Similarly, NF-κB reporter cells have been treated with MSC-EVs, and their increase in luminescence has been detected for NF-κB activation (Goloviznina et al., 2016). Similar luciferase-reporter cell lines for the notch, hedgehog, and Wnt signaling are commercially available (Accegen) to test whether specific cell-derived EVs can stimulate these singling pathways. Further, various platforms such as nCounter®-Stem Cell Characterization Panel (NanoString Technologies), that contain a panel of around 770 genes is available to evaluate stem cell viability and functionality. Such platforms need to be developed for screening the potential of biomolecules present in EVs. Alternately, conventional proteomics and miRNA sequencing are also available for detailed cargo profiling in the EVs of interest.

The small size and short life span of EVs make it more challenging to track them *in vivo*; however, *ex vivo* membrane labeling of EVs or labeled EVs (CD63-GFP) from reporter-cell lines or mice (Luo et al., 2020) are an alternate. Tracking EVs with these strategies will reveal their bio-distribution *in vivo* and help design better dosing strategies to target radiation-induced injury.

In situations such as accidental exposure to radiation (DiCarlo et al., 2017), the EVs need to be administered within 24–48 h to rescue bone marrow. In such a scenario, scaled-up and cryopreserved EVs should to be available to the patients in a short period of time. Therefore, EV-biobanks operated with clinical good manufacturing practice (cGMP) standards under FDA regulations are necessary to support the development of EV-therapeutics.

Biogenesis of EVs is a cellular response to physiological stimuli; therefore, *in vitro* culture conditions and treatments such as growth factors can influence the quantitative and qualitative production of EVs. For instance, serum-free culture or usage of exosome-free serum is often used to culture cells for EV production, which might alter the natural EV production threshold. On the other hand, parameters such as pH, temperature, and cryopreservation conditions can alter the EV uptake by cells (Cheng et al., 2019). Therefore, optimization of these parameters is needed to improve the purity and yield of EVs and their efficacy.

### Standardization

Heterogeneity in the EV sub-populations poses them as challenging for developing as therapeutics. Simultaneously, their diversity in containing various protein, nucleic acid, and lipid in the cargo makes them attractive for targeted delivery with multi-functional activity. Thorough characterization of EVs according to MISEV guidelines and reproducibility in the generation of EVs of interest is essential. GMP grade methodologies for EV preparation need to be developed for clinical translation. To further develop EVs as radiation MCM, the quality of cell source, cell culture, and method of EV preparation need to be standardized and reproducible across respective fields, followed by the *in vitro* and *in vivo* functionality assessment.

With the growing interest in the EVs for research and for clinical use, it is extremely important for universally acceptable methods and standards. ISEV community and its tools such as EV-TRACK and EV-METRIC are supporting EV-based research laboratories with centralized knowledge of EVs (Van Deun et al., 2017) (Royo et al., 2020). Researchers can submit their experimental data related to the method of isolation, characterization, and analysis from their projects to obtain constructive input and validation from EV-TRACK. In addition, workshops, conferences, and interactive scientific discussions organized by ISEV are enhancing the collaborative network of the EV research community, share, expand the knowledge between research laboratories around the globe, and collectively troubleshoot the challenges in EV research.

### Expansion

Large-scale production of EVs is dependent on the type of cell source and method of isolation. Scaling up from T-flasks to bioreactors can increase the production of EVs. However, these changes in the culturing environment might influence cells and thereby change the characteristics of EVs. Therefore, these parameters need to be standardized in the scale-up process. The quality of EVs and their bioactivity need to be confirmed after the scale-up. Methods of EV isolation include but are not limited to differential/density-gradient ultracentrifugation, tangential flow filtration, bind/elute chromatography, size-specific separation, and immunolabeling-based EV selection. There is a unique principle and advantage for each of these methods. Ultracentrifugation is the most popularly used (Royo et al., 2020) and considered the gold standard; however, it might not be suitable for the purification of EVs on a large scale. Therefore, a combination of techniques might be an alternate strategy to obtain pure EVs.

### Storage

Another major challenge in EV research is their storage. Literature suggests storing EVs at +4°C for a few weeks. EVs can be stored long-term at −80°C either in PBS or with cryo-protectants such as DMSO, Trehalose (Bosch et al., 2016), and glycerol (Jeyaram and Jay, 2017). For cryopreservation of various cell types, commercial reagents such as CryoStor, and NutriFreez are available. More GMP suitable cryopreservation reagents need to be developed for EV preservation. On other hand freeze-dried EVs can be a greater source for long-term storage and therapeutic applications (El Baradie et al., 2020).

### Safety and Side Effects

In cell transplant studies, the *in vivo* microenvironment signals might influence viability, proliferation, and functionality of the transplanted cells. This might not be an issue in EV therapeutics since they do not replicate or differentiate and are short-lived. However, EVs that are naturally produced by a cell type are not necessarily specific to one tissue. Also, the multi-factorial bioactivity of EVs might cause side effects, a safety concern for targeted therapy. Another function of EVs secreted from the cells is a self-defense mechanism to maintain cellular homeostasis by removing the toxic molecules from the cells (Wallis et al., 2020).

To enhance a more targeted approach and minimize side effects, EVs can be locally administered to a specific tissue, such as intra-glandular injections at the site of injury. Alternately, engineered cells that produce tissue-specific EVs could be used.

EVs obtained from genetically engineered and immortalized cell lines might differ from naturally occurring EVs. Therefore, they need to be verified for purity, characterization, mechanism of action, *in vivo* biodistribution, and bioactivity. Genetically engineered cell-derived EVs might carry oncogenic remnants; therefore, they need to be verified for immunogenic and tumorigenic side effects.

There is a growing interest in using EVs as natural drug-delivery vehicles. Physical and chemical methods are used for drug-loading into the EVs. Hence, any immunogenic effect arising from these methodologies in the processing of EVs needs to be verified.

Overall, EVs are no exception with respect to having safety regulations for therapeutic application. Aspects such as production methods, purification, dosing, storage, and administration are important to check points for safe EV therapeutics.

## Conclusion

**FIGURE 4:**
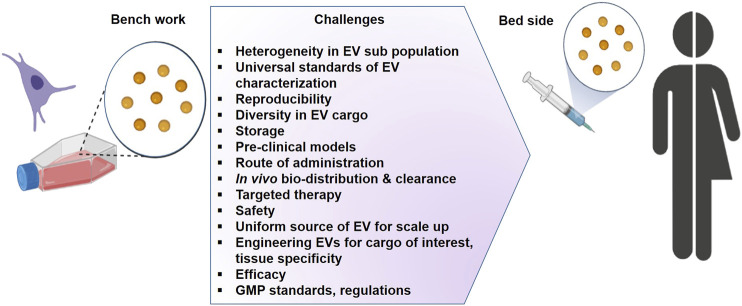
The challenges for the development of extracellular vesicles as therapeutics (Graphic adapted from BioRender.com).

An overview of the literature clearly indicates that research on EVs suffers from limitations. A number of technical points remain to be urgently clarified as summarized in Figure 4. However, the complexity of EVs heterogeneity and functions has to be taken into account. One of the most urgent challenges is to set up methods to characterize separately each kind of EVs in order to precisely define their individual cargoes and functions. The first challenge is how to define and measure EVs in a reproducible manner, their large scale production and purification for their use in case of mass casualties. In addition, understanding the molecular mechanisms governing EV formation, release, and clearance, as well as those involved in cell–cell communication, will enable us to envision new therapeutic strategies for favoring tissue repair. As described in this review several biological features provide EVs as an attractive tool for regenerative medicine. The advancement of translational research directed toward treating battlefield injury will have multiple cross over opportunities for their applications within the population. They will surely be a part of innovative therapeutic interventions as soon as few technical barriers are solved.

The adaptability of BMMSC-, EC- and Mɸ-derived EVs exhibit a remarkable capacity to adapt to the requirements of the damaged tissue in which the vesicles integrate and provide a promising option to address for the medical field after radiation exposure and complication of radiotherapy in order to support a personalized treatment. MSC therapy has established an extensive safety profile in the clinical setting and compared to the other cell types such as the endothelial progenitor cells, they present important advantage related to their isolation, culture and high scale production. Thus, in case of a radiological or nuclear event, the use of MSC-EV approach will provide a promising option to address the unmet needs that are critically important in the medical management of mass casualties.

## Glossary

ARS: Acute radiation syndrome
BMASC: Bone marrow-derived adherent stromal cell
BMMSC: Bone marrow mesenchymal stem cells
BMM: Bone marrow macrophage
BV/TV: Bone volume fraction
CCL2: Chemokine (C-C motif) ligand 2
DAMP: Damage-associated molecular patterns
DEARE: Delayed effects of acute radiation exposure
DSB: double-strand breaks
DSS: Dextran sulfate sodium
EC: Endothelial cell
EC-EVs: Endothelial cell-extracellular vesicles
ECFC: Endothelial colony-forming cells
ERK1/2: Extracellular signal-regulated protein kinase
EM: Electron microscopy
EMMPRIN: Extracellular matrix metalloproteinase inducer
EV: Extracellular vesicle
FDA: Food and drug administration
G-CSF: Granulocyte colony-stimulating factor
GM-CSF: Granulocyte-macrophage colony-stimulating factor
Gy: unit of radiation
Gray =: 100 rads
GMP: Good manufacturing practice
HSCs: Hematopoietic stem cells
HUVEC: Human umbilical cord vein endothelial cells
ICAM1: Intercellular adhesion molecule 1
iPSC: induced pluripotent stem cells
IL: Interleukin
ISC: Intestinal Stem Cells
ISEV: International society of extracellular vesicles
Lgr5: Leucine-rich repeat-containing G-protein coupled receptor 5
MAPK: Mitogen-activated protein kinase
Mɸs: Macrophages
MCM: Medical countermeasure
MISEV: Minimal information for studies on extracellular vesicles
MM6: Mono-Mac-6
MSC: Mesenchymal Stem Cells
NF-κB: nuclear factor kappa-light-chain-enhancer of activated B cells
NIH: National Institute of Health
PTEN: Phosphatase and Tensin Homolog
RIBE: Radiation-induced bystander effect
RILD: Radiation-induced liver disease
ROS: reactive oxygen species
SASP: Senescence-associated secreted phenotype
SH-SY5Y: human neuroblastoma cells
SOD: Super oxide dismutase
SSB: Single strand breaks
TCF/LEF: T-cell factor/lymphoid enhancer factor
TNF-α: Tumor necrosis factor-alpha
TSG101: Tumor susceptibility gene 101
Plau/uPA: urokinase-type plasminogen activator
VCAM1: Vascular cell adhesion molecule 1
VE-cadherin: Vascular endothelial-cadherin
WBI: Whole-body irradiation
